# Cultural adaptation to quechua and psychometric analysis of the patient health questionnaire (PHQ-9) in a peruvian population

**DOI:** 10.17843/rpmesp.2023.403.12571

**Published:** 2023-09-26

**Authors:** Julio Cjuno, Félix Julca-Guerrero, Yulisa Oruro-Zuloaga, Frinee Cruz-Mendoza, Admirson Auccatoma-Quispe, Heber Gómez Hurtado, Frank Peralta-Alvarez, Juan Carlos Bazo-Alvarez

**Affiliations:** 1 Universidad Cesar Vallejo, Faculty of Medicina, Piura, Peru. Universidad Cesar Vallejo Universidad Cesar Vallejo Faculty of Medicina Piura Peru; 2 Universidad Nacional Santiago Antúnez de Mayolo, Huaraz, Peru Universidad Nacional Santiago Antúnez de Mayolo Universidad Nacional Santiago Antúnez de Mayolo Huaraz Peru; 3 Universidad Peruana Unión, Professional School of Psychology, Lima, Peru. Universidad Peruana Unión Universidad Peruana Unión Professional School of Psychology Lima Peru; 4 Escuela de Educación superior pedagógico José Salvador Cavero Ovalle, Ayacucho, Peru. Escuela de Educación superior pedagógico José Salvador Cavero Ovalle Ayacucho Peru; 5 Educational Institution N° 38848 Pucara-Uchuraccay, Ayacucho, Peru Educational Institution N° 38848 Pucara-Uchuraccay Ayacucho Peru; 6 Universidad Tecnológica del Perú, Chimbote, Peru. Universidad Tecnológica del Perú Universidad Tecnológica del Perú Chimbote Peru; 7 Institute for Psychosocial and Educational Research, Training and Development PSYCOPERU, Chimbote, Peru. Institute for Psychosocial and Educational Research, Training and Development PSYCOPERU Chimbote Peru; 8 Universidad Sedes Sapientiae, Lima, Peru. Universidad Sedes Sapientiae Lima Peru; 9 Universidad Privada Norbert Wiener, Lima, Peru. Universidad Privada Norbert Wiener Universidad Privada Norbert Wiener Lima Peru; 10 Research Department of Primary Care and Population Health, University College London, London, United Kingdom University College London Research Department of Primary Care and Population Health University College London Londres United Kingdom

**Keywords:** Patient Health Questionnaire, depression, Indigenous Peoples, Psychometrics

## Abstract

**Objective:**

. To translate and culturally adapt the Patient Health Questionnaire (PHQ-9) to three varieties of Quechua and analyse their validity, reliability, and measurement invariance.

**Materials and methods:**

. 1) Cultural adaptation phase: the PHQ-9 was translated from English into three variants of Quechua (Central, Chanca, Cuzco-Collao) and translated again into English. Then, experts and focus groups allowed the translations to be culturally adapted. 2) Psychometric phase: the unidimensionality of the adapted PHQ-9 was evaluated by using Confirmatory Factor Analysis (CFA), reliability was evaluated by internal consistency (Alpha and Omega), and measurement invariance according to Quechua varieties and sociodemographic variables was evaluated by using CFA, multigroups and MIMIC models (Multiple Indicator Multiple Cause).

**Results:**

. Each of the adaptations of the PHQ-9 to the three Quechua varieties reported clear and culturally equivalent items. Subsequently, data from 970 Quechua-speaking adult men and women were analyzed. The general one-dimensional model reported an adequate fit (Comparative fit index = 0.990, Tucker-Lewis index = 0.987, Standardized root mean squared residual= 0.048, Root mean squared error of approximation=0.071); each of the Quechua varieties also showed an adequate fit. Reliability was high for all varieties (α = 0.865 - 0.915; ω = 0.833 - 0.881). The results of the multigroup CFA and MIMIC models confirmed measurement invariance according to Quechua variant, sex, residence, age, marital status and educational level.

**Conclusions:**

. The PHQ-9 adaptations to Central Quechua, Chanca and Cuzco-Collao offer a valid, reliable and invariant measurement, confirming that comparisons can be made between the evaluated groups. Its use will benefit mental health research and care for Quechua-speaking populations.

## INTRODUCTION

Depression is a frequent mental disorder, and is caused by complex interactions between social, psychological and biological factors ^1^. According to the World Health Organization, more than 5% of the world population had depression in 2021 ^2^, increasing by 25% during the COVID-19 pandemic ^3^. In Peru, of 57,446 respondents, 60.1% of 9383 persons with a mental health diagnosis living in the Peruvian highlands and coast, with the exception of Metropolitan Lima, had depressive symptoms during the pandemic ^4^. Another Peruvian study, that evaluated 31,996 participants, found that living in the highland region was an important factor for the increase in clinically relevant depressive symptoms, reporting 9.0% of moderate to severe cases, compared with 5.8% in other regions ^5^.

The Patient Health Questionnaire (PHQ-9) is a psychometric instrument designed to assess depressive symptoms according to the criteria of the Diagnostic and Statistical Manual of Mental Disorders - IV ^6^. Originally written in English with a single factor ^7^, it is widely used in clinical practice and research internationally ^8^. It has been adapted to more than 18 languages in 24 countries ^9^, such as French ^10^, Mandarin Chinese ^11^, Spanish ^12^, Russian ^13^, German ^14^, Norwegian ^15^, Farsi ^16^, Lithuanian ^17^^)^ and even Kinyarwanda ^18^. The Spanish version of this instrument has shown good properties among Spanish speakers in Peru ^1^.

In a previous study on the Peruvian version of the PHQ-9 (in Spanish) ^1^, we reported evidence of measurement invariance according to socio-demographic variables: sex, age, educational level, socioeconomic status, marital status and area of residence (rural/urban). Such evidence allows comparisons between different groups (according to the categories of these variables), ensuring that PHQ-9 results indicate a similar underlying experience of depressive symptoms across these groups (e.g., men and women) ^19^. This invariance assessment has not been applied to any version of the PHQ-9 in Quechua yet. Without invariance analysis, there is no guarantee that the PHQ-9 measures depression in the same way in all Quechua-speaking groups, hence its necessity ^20^.

However, there is still no Quechua version of the PHQ-9 for the Peruvian population. Peru has 3,799,780 inhabitants who have Quechua as their first language, representing 13.6% of the census population in 2017 ^21^. People ethnically self-identified as Quechua may perceive and express depressive symptoms differently than a Spanish speaker, which makes it difficult to use the PHQ-9 in Spanish. On the other hand, Quechua is a family of languages and presents much dialectal variation ^22^. The main varieties by number of speakers and geographical extension are: Central Quechua (CQ), Ayacucho-Chanka Quechua (ACQ) and Cuzco-Collao Quechua (CCQ), which present many differences ^23^^,^^24^. Phonologically, CQ has 24 phonemes, ACQ has 19 phonemes and CCQ has 28 phonemes. Morphologically, for example, the progressive action and the first person “I am going”; in QC is marked with -yka and -V (aywa-yka-a), in ACQ it is marked with -chka and -ni (ri-chka-ni) and in CCQ it is marked with -sha and -ni (ri-sha-ni). Likewise, lexically, the translation of the words “yellow” and “accelerate” is *qallwash* and *wip* in CQ, *hillu* and *utqay* in ACQ and *q’illu* and *utqhay* in CCQ, respectively ^24^. Thus, the linguistic variation is wide and; consequently, mutual intercomprehension among speakers of the various variants of Quechua is very difficult and almost impossible; therefore, a single survey could not be conducted using only one of the varieties. The *ad hoc* solution typically adopted by local users is to have a third bilingual person “translate” the questions and answers to the questionnaire (e.g., a younger family member with a different worldview than the person being assessed). However, this practice is not recommended because it introduces noise into the assessment ^25^. In Peru, most health personnel are not fluent in Quechua as a first or second language, as mentioned by Montesinos-Segura *et al*. ^26^ in the discussion of their study. In this sense, an appropriate assessment of depressive symptoms by health personnel in the general Quechua-speaking population is very difficult at present, given this inherent language limitation.

Therefore, the aim of our study was to translate and culturally adapt the PHQ-9 to three varieties of Quechua, and to analyze the internal structure validity, reliability and measurement invariance (by Quechua variants and sociodemographic variables) of the adapted instrument.

**KEY MESSAGES**

Motivation for the study. Peru is the country with the largest Quechua-speaking population in South America, but it does not have an instrument to assess depression culturally adapted to Quechua populations.

Main findings. A valid and reliable version of the PHQ-9 was obtained for use in Quechua-speaking populations of the Central, Chanca and Cuzco-Collao varieties.

Implications. This new version could be implemented in national health surveys and community mental health centers for screening and evaluation of depressive symptoms.

## MATERIAL AND METHODS

### Design and context

Instrumental study ^27^, carried out in four departments of Peru (Ancash, Ayacucho, Puno and Cuzco) according to the variety of Quechua. We selected adults from the departments of Puno and Cuzco, located in southwestern Peru in order to evaluate the Cuzco-Collao variety. Puno has 538,127 (57.0%) and Cuzco has 709,892 (74.7%) Quechua speakers ^28^; both departments are characterized by commercial activities, livestock farming and tourism due to their richness in historical and archaeological sites. To evaluate the variety of Quechua Chanca, we selected inhabitants of the department of Ayacucho, which has 389,045 (81.2%) Quechua speakers, mostly dedicated to commerce, livestock farming and agriculture ^28^. In order to evaluate the variety of Central Quechua, we selected inhabitants of the department of Ancash, which has 289,172 (34.0%) Quechua speakers, mostly engaged in commerce, agriculture and tourism ^28^.

### Cultural adaptation phase

*Translation*

We used the forward translation and back translation methods during the translation process ^27^. The original version of the PHQ-9 (English) was translated directly into each variety of Quechua (Central, Cuzco-Collao, Chanca) by two independent translators, who are native Quechua speakers of each variety and with advanced knowledge of English. Once the translation for each variety was completed, the two translators and two native Quechua-speaking researchers met to discuss the differences in the translations. Once the discrepancies were resolved and the translations unified, we proceeded with the back translation (Quechua to English) of the three Quechua variants of the PHQ-9. This process was carried out by two translators who had English as their native language and advanced knowledge of Quechua for each variant. Once the back translations were completed, the translators for each variant met with two study researchers to verify the back translation along with the first translation, fine-tuning details and giving their approval to the final version for the three languages.

### Cultural adaptation to Quechua contexts

By applying the Delphi method, we created a cultural adaptation form, which can be found at: https://doi.org/10.5281/zenodo.8312191. This form included some open questions for uncommon words in Quechua such as “depression” or “without hope”. We also sought to consult on the change of “reading newspaper” to “listening to radio”, this change involves the PHQ-9 response categories and the relationship between the PHQ-9 in Quechua with the DSM-V for the diagnosis of major depression. The Central Quechua version of the PHQ-9 was reviewed by two Quechua-speaking psychologists, who had at least three years of experience in the care of Quechua-speaking patients with depression in the provinces of Ancash; one of them had a master’s degree and the other a bachelor’s degree in psychology. The Cuzco-Collao Quechua version was evaluated by five Quechua-speaking psychologists, all with professional degrees and at least one year of experience in the care of Quechua-speaking patients with depression in Puno or Cuzco. The Chanca Quechua version was evaluated by two psychologists with professional degrees and at least one year of experience in the care of Quechua-speaking patients with depression in the provinces of Ayacucho. The interaction between each expert and the research team took place in two (Central) and four rounds (Cuzco-Collao and Chanca) of mailings. In addition, we assessed the improvements (based on the recommendations of the experts) using indicators of relevance, representativeness, clarity and cultural equivalence on a scale of 0 to 3 (where 3 was the best rating). Once all the experts rated the improvements in all indicators with a score of 3, we held a virtual meeting with the research team to evaluate the suggestions and reach a consensus.

**Figure 1:**
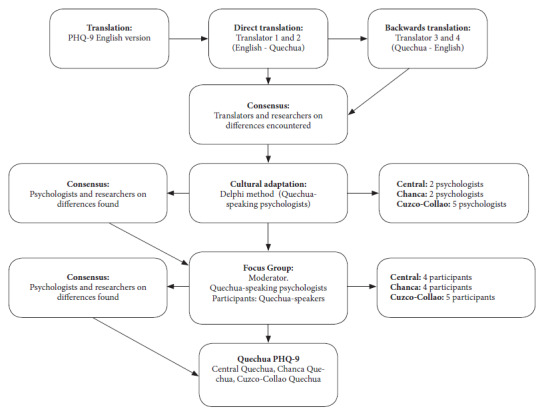
Flow chart of the translation and cultural adaptation of the PHQ-9.

Subsequently, a focus group (via Zoom) was organized for each variety of Quechua. The moderator for each variety was a Quechua-speaking psychologist proficient in qualitative methods. The meetings lasted approximately 60 minutes. First, we asked participants to answer the PHQ-9 in Quechua through an online version shared via Google Forms. Then, the moderator invited the participants to provide their opinion on the clarity and comprehension of the items in a language that was common and simple for Quechua speakers. Five Quechua speakers participated (three women and two men) in the focus group for the Cuzco-Collao variety; four people participated (two women and two men) for the Quechua Chanca variety; and four adults participated (three men and one woman) in the focus group for the Central Quechua variety. We considered, for the three varieties of Quechua, that the participants be bilingual (speak Quechua and Spanish) and over 18 years of age (Figure 1). The focus group participants provided a favorable opinion regarding the clarity and comprehension of the PHQ-9 in the three variants of Quechua, confirming that this adaptation is clear and in accordance with their cultural context.

### Psychometric analysis phase

*Participants*

The final version of the instrument, obtained after the previous phase, was applied to a sample of 970 adults selected by non-probabilistic convenience sampling, ensuring that for each variety of Quechua we had no less than 200 participants. This number is even higher than the recommended standard for factor analyses (20 participants per item and considering that there are 9 items, the minimum size would be 180 participants) and reported as adequate in simulation studies ^29^. It also complies with the minimum size calculated for factor analysis (n=218, see: https://doi.org/10.5281/zenodo.8312191). Six surveyors participated in the data collection, they presented the study to the participants, and once informed consent was obtained, they requested the participants to complete the survey via Google Forms. The collection was carried out in those towns and cities with the greatest Quechua presence. Men and women, over 18 years of age, living in urban and rural settings, located in three regions (according to Quechua variant) were included. We included inhabitants of the departments of Puno and Cusco (n=525) to evaluate the Cuzco-Collao variant, inhabitants of Ayacucho (n=226) to evaluate the Chanca variant, and inhabitants of the department of Ancash (n=219) to analyze the Central Quechua variant. All participants were bilingual Quechua speakers (Quechua and Spanish), with sufficient academic background to read Quechua (at least incomplete primary school). Those who spoke a variety of Quechua other than the three studied were excluded.

*Instrument*

The PHQ-9 consists of 9 items corresponding to DSM-IV depressive symptoms ^6^. The response options evoke the frequency of occurrence of such symptoms in the last two weeks, considering the following Likert-type scale: 0 = not at all, 1 = several days (1-6 days), 2 = most days (7-11 days), 3 = almost every day (12 days or more). The Spanish version of the PHQ-9 has demonstrated validity (e.g., goodness of fit as a unidimensional measure: CFI [comparative fit index] = 0.936; RMSEA [root mean square error of approximation] = 0.089; SRMR [standardized root mean square residual] = 0.039), as well as adequate reliability (α = ω = 0.87) in the Spanish-speaking Peruvian population ^1^.

*Covariates*

They were used to characterize the population, as well as to study the measurement invariance of the unidimensional model according to age (in years), sex (female, male), education (incomplete primary, complete primary, incomplete secondary, complete secondary, incomplete higher, complete higher), marital status (single, married/cohabiting, divorced/separated, widowed) and place of residence (rural/urban).

*Procedure*

For data collection, one interviewer for the Central Quechua variety, two interviewers for the Chanca Quechua variety, and three interviewers for the Cuzco-Collao Quechua variety were trained in the use and application of the instrument. All interviewers were third- or fourth-year psychology students. The interviewers identified WhatsApp groups of parents from educational institutions, Christian churches and groups of peasant community associations to whom they presented the survey in Google Forms format. Data collection began in February and ended in June 2022.

*Statistical analysis*

Relative and absolute frequencies were used during descriptive analysis ^30^. Subsequently, we conducted a Confirmatory Factor Analysis (CFA) of the unidimensional model (its unidimensionality was verified in Peru in a representative sample of 30,449 individuals: https://doi.org/10.5281/zenodo.8312191) using a WLSMV estimator (weighted robust squares with adjusted mean and variance), as we did previously ^1^. In addition, the WLSMV is an unbiased estimator for items with ordinal responses and non-normal distribution ^31^. We report the standardized betas of the model and standard goodness-of-fit measures: the chi-square (X^2^) for the model versus baseline, considering values <3 as acceptable; the CFI, which is adequate when >0.90; the TLI (Tucker-Lewis index), which is acceptable with values >0.90. Likewise, the SRMR and RMSEA, were considered adequate with values ≤ 0.08 ^32^.

We assessed measurement invariance across groups defined by Quechua variants, sex and location using a multigroup CFA. The change in CFI (ΔCFI) and RMSEA (ΔRMSEA) was used as the main criterion to compare models with more constraints versus models with fewer constraints. Models first assumed configurational invariance (i.e., similar factor structure across groups) as the base model, moving up to metric invariance (i.e., similar factor loadings and factor structure across groups), strong invariance (i.e., similar thresholds, factor loadings, and factor structure across groups), and strict invariance (i.e., similar residual variances of items, thresholds, factor loadings, and factor structure across groups). Between each model, we examined whether ΔCFI < 0.01 or ΔRMSEA < 0.01 in order to establish whether the more restricted model was appropriate ^30^.

Alternatively, MIMIC (Multiple Indicators and Multiple Causes) models were adjusted for the evaluation of measurement invariance according to age, marital status and educational level (variables for which the multigroup CFA was not feasible). We assessed the invariance of indicator intercepts and mean differences of latent dimensions, all across groups according to these covariates. We preferred to use ∆CFI rather than ΔX^2^ because the former is not affected by sample size or model complexity ^32^. For multigroup CFA, we performed a sensitivity analysis using the method recommended by Yoon and Lai ^33^ for dissimilar sample sizes. In fact, each covariate was evaluated separately, comparing two types of models for each of them: 1) a saturated version where the covariate explains all the observed items, but not the latent dimensions, and 2) a version of the invariant intercept model where the covariate explains all the latent dimensions, but not the items. Similarly, the above fit indices are reported and interpreted.

Finally, reliability was determined using Cronbach’s Alpha ^34^^)^ and McDonald’s Omega ^35^^)^ coefficients. To ensure reproducibility, the main codes of the analysis can be found at: https://github.com/JCBAZO/R-PHQ9-Quechua. All analyses were performed in R Studio version 4.0.4, with the packages “lavaan” ^36^, “lavaan.survey” ^37^ “semTools” ^38^, “semPlot” ^39^^)^ and “Psych” ^40^.

*Ethical Considerations*

This study was evaluated and approved by the Ethics Committee of the Faculty of Health Sciences of the Universidad Peruana Unión with report number 2022-CE-FCS-UPeU-059. The instrument was applied as a self-report in virtual format (Google Forms), designed to present the informed consent first, so only those who agreed to participate in the study accessed the survey. Authorization was requested from the copyright owner of the original instrument (Pfizer) via e-mail, who authorized its use and adaptation.

## RESULTS

### Cultural adaptation phase

The experts and the research team interacted until achieving the highest rating of appropriateness for each item with respect to its cultural adaptation (3 on a range of 0 to 3), highlighting its relevance, representativeness, clarity and cultural equivalence. Additionally, they provided valuable suggestions which are mentioned below.

Some recommendations contributed to improve the adaptation of the word “depression”, which was initially translated as “*llaqui*”. Experts suggested to add “*sinchi*”, which gives a greater intensity, leaving “*sinchi llakisqa*” (a lot sadness) as the best understanding of depression (item 2). Regarding the expression “without hope”, the experts recommended implementing the question as “*manañan q’anchay karqanchu kausayñiykipi / yanqallañan kausaranki*” (item 2). For contextual reasons, the daily activity “reading the newspaper” was changed to “listening to the radio”, because the latter is the most used media in the three regions (item 7).

The response options (Likert-type) of the translated PHQ-9 also required special attention on the part of the judges and focus group participants. In particular, the category “almost every day” presented problems of clarity in its translation. The team’s final recommendation was to use the expressions *“Mana hayk’aqpas”, “Wakin p’unchawkunalla”, “Ashka p’unchawkuna”, “Yaqa llapa p’unchawkuna”,* representing the Spanish equivalent of “Never”, “Some days”, “Several days”, “Almost every day”.

### Psychometric analysis phase

*Characterization data*

Of the 970 bilingual participants, 560 (57.7%) were female, 580 (59.8%) were between 18 and 30 years old, 621 (64%) reported being currently at or having completed college, 577 (59.5%) were single, and 614 (63.3%) lived in an urban area (Table 1).

**Table 1:** Characteristics of the study participants.

Characteristics	Total		Central		Chanca		Cuzco-Collao	
	(n=970)		(n=219)		(n=226)		(n=525)	
	n	(%)	n	(%)	n	(%)	n	(%)
Sex								
Women	560	57.7	122	55.7	141	62.4	297	56.6
Men	410	42.3	97	44.3	85	37.6	228	43.4
Age (years)								
18-30	580	59.8	115	52.5	178	78.8	287	54.7
31-40	166	17.1	41	18.7	22	9.7	103	19.6
41-50	120	12.4	23	10.5	18	8.0	79	15.1
51-68	104	10.7	40	18.3	8	3.5	56	10.6
Education								
Incomplete primary school	42	4.3	0	0.0	9	4.0	33	6.3
Complete primary school	38	3.9	4	1.8	2	0.9	32	6.1
Incomplete secondary school	40	4.2	8	3.7	8	3.5	24	4.6
Complete secondary school	229	23.6	35	16.0	19	8.4	175	33.3
Incomplete higher education	325	33.5	75	34.2	114	50.4	136	25.9
Complete higher education	296	30.5	97	44.3	74	32.8	125	23.8
Civil status								
Single	577	59.5	131	59.8	167	73.9	279	53.1
Married/Cohabitant	335	34.5	68	31.1	53	23.4	214	40.7
Divorced/Separated	36	3.7	16	7.3	4	1.8	16	3.1
Widow	22	2.3	4	1.8	2	0.9	16	3.1
Residence								
Urban	614	63.3	155	70.8	144	63.7	315	60.0
Rural	356	36.7	64	29.2	82	36.3	210	40.0

*Validity of internal structure*

**Figure 2:**
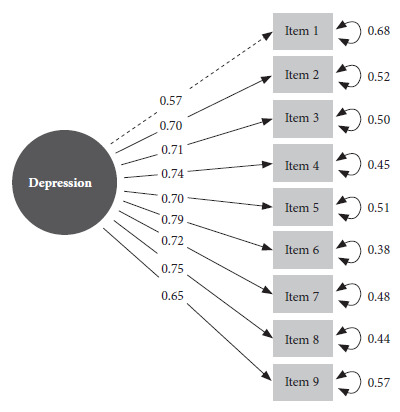
General measurement model of the PHQ-9 with betas and standardized errors (N=970).

The three final instruments (one for each variety of Quechua) were validated independently. The single-factor model reported adequate goodness-of-fit values for the three varieties of Quechua (Central, Chanca and Cuzco-Collao), as well as for the total sample (CFI= 0.990; TLI= 0.987; SRMR=0.048; RMSEA= 0.071) (Table 2). On the other hand, the sensitivity analysis for the multigroup CFA reported results similar to those shown here. The only latent factor of the measurement model (depression) loaded a minimum of λ = 0.57 and a maximum of λ = 0.79 to the PHQ-9 items (Figure 2).

**Table 2:** Goodness of fit of the unidimensional PHQ-9 measurement model and reliability, of the total sample and by Quechua variants.

Goodness-of-fit index	Total (N=970)	Central (n=219)	Chanca (n=226)	Cuzco-Collao (n=525)
χ^2^ (36)	13513	2381	2653	10041
CFI	0.990	0.968	0.998	0.995
TLI	0.987	0.958	0.997	0.994
SRMR	0.048	0.082	0.029	0.042
RMSEA	0.071	0.112	0.048	0.058
Alpha	0.895	0.865	0.877	0.915
Omega	0.861	0.834	0.833	0.881

X^2^: Chi-square, X^2^(df): for model versus baseline, df: degrees of freedom, CFI: comparative fit index, TLI: Tucker-Lewis Index, SRMR: standardized root mean square residual, RMSEA: root mean square error of approximation.

*Reliability*

The PHQ-9 reported good reliability for all Quechua varieties, with Cronbach’s Alpha values between 0.865 and 0.915, and with Omega values between 0.833 and 0.881 (Table 2).

*Measurement invariance*

The results of the multigroup CFA confirm the invariance of measurement according to Quechua variants, sex and place of residence (Table 3). The configural model showed good fit to the data (CFI = 0.935) for the three Quechua variants. After confirming configural invariance, metric invariance was proven with ΔCFI = 0.001 and ΔRMSEA=0.007 (both <0.01). Then, the values of ΔCFI=0.018 and ΔRMSEA=0.005 demonstrated strong invariance, with at least the latter being <0.01. Finally, strict invariance was confirmed with ΔCFI=0.009 and ΔRMSEA=0.001. The results and interpretation were similar for the invariance models according to sex and residence (Table 3).

**Table 3:** Fit indices of the measurement invariance tests between groups according to Quechua variants, sex and place of residence (N=970).

Variable	PHQ-9 (Internal structure)	Invariance (Model)	X^2^	df	p-value	CFI	Δ CFI	RMSEA	Δ RMSEA
Quechua variant	Unidimensional	1. Configural	207.1	81	-	0.965	-	0.100	-
		2. Metric	296.3	97	<0.001	0.964	0.001	0.093	0.007
		3. Strong	423.2	131	<0.001	0.947	0.018	0.097	0.005
		4. Strict	523.9	149	<0.001	0.938	0.009	0.098	0.001
Sex	Unidimensional	1. Configural	182.0	54	-	0.963	-	0.098	-
		2. Metric	192.0	62	0.683	0.971	0.008	0.082	0.017
		3. Strong	201.3	79	0.539	0.970	0.001	0.074	0.008
		4. Strict	212.1	88	0.443	0.971	0.002	0.068	0.006
Residence (urban/rural)	Unidimensional	1. Configural	180.5	54	-	0.962	-	0.098	-
		2. Metric	220.7	62	<0.001	0.962	0.000	0.091	0.007
		3. Strong	214.3	79	1.000	0.965	0.003	0.078	0.013
		4. Strict	241.8	88	0.005	0.963	0.002	0.075	0.002

X^2^: Chi-square, df: degrees of freedom, CFI: comparative fit index, RMSEA: root mean squared error of approximation, Δ: difference, X^2^(df): for model versus baseline (Satorra-Betler).

The results of the MIMIC models confirmed the invariance by age, marital status and educational level (Table 4). The CFI and TLI values were >0.98 for all the variables, while the SRMR and RMSEA values were <0.08. Likewise, the absolute values of ΔCFI, ΔTLI and ΔRMSEA were <0.01.

**Table 4:** Goodness of fit of the MIMIC models for the PHQ-9 (N=970)

Covariable	Model	CFI	TLI	RMSEA	SRMR	Δ CFI	Δ TLI	Δ RMSEA
Age	Saturated MIMIC	0.990	0.984	0.071	0.048	-	-	-
	MIMIC of invariant intercept	0.990	0.987	0.063	0.048	0.000	0.003	-0.008
Civil status	Saturated MIMIC	0.990	0.984	0.070	0.048	-	-	-
	MIMIC of invariant intercept	0.990	0.987	0.064	0.048	0.000	0.003	-0.006
Education	Saturated MIMIC	0.991	0.985	0.069	0.047	-	-	-
	MIMIC of invariant intercept	0.990	0.987	0.064	0.047	-0.001	0.002	-0.005

MIMIC: Multiple Indicators and Multiple Causes, CFI: comparative fit index, TLI: Tucker-Lewis index, SRMR: standardized root mean square residual, RMSEA: root mean square error of approximation, Δ: difference. Comparisons (Δ) were made between the saturated MIMIC model and the MIMIC of invariant intercept model for each covariate studied.

## DISCUSSION

This is the first study on the cultural adaptation to Quechua of the PHQ-9, a standardized instrument used internationally to assess depressive symptoms. After completing a back-and-forth translation (English-Quechua-English) for the three variants of Quechua, we carried out the cultural adaptation with the help of expert judges and members of the target population, all Quechua speakers. The adapted version offers a unidimensional, reliable and invariant measurement across groups according to Quechua variant, gender, residence, age, marital status and educational level. This invariance confirms that comparisons can be made with the PHQ-9 Quechua measurements across the aforementioned groups. The final instruments can be found in the Supplementary Material.

This cultural adaptation opens new possibilities for the assessment of depression in the Peruvian Quechua-speaking population, both for research and clinical purposes. Previously, a narrative review that searched Pubmed, Web of Science and Scopus, included studies in English and/or Spanish, found seven studies that assessed depression in Quechua-speaking populations ^41^. Only two of these studies used an instrument translated and adapted into Quechua, the Hopkins Symptoms Checklist (HSCL-25); however, this adaptation only considered one variant: Ayacuchan Quechua ^42^. The distinction of variants is essential to be able to cover larger groups of people in the different regions of Peru, otherwise, the problem of having to resort to a third person to translate or interpret the questions and answers at the time of application persists. The PHQ-9 adapted to three versions of Quechua reduces the need for such support, facilitating assessment both in research (e.g., DHS-type health surveys) and in clinical settings (e.g., standardized instrument required according to DSM-V diagnostic criteria).

The adapted instruments have psychometric properties similar to those of the original PHQ-9 and the one validated for Spanish-speaking Peruvians. In both cases, the internal structure of the PHQ-9 was determined to be unidimensional, i.e., a single latent representing depression and expressed through each of the nine symptoms assessed by the instrument ^1^^,^^7^. Internationally, recent systematic evidence supports the unidimensional model across cultures ^43^, with such acceptance that the debate is now focused on defining the most accurate cut-off points for a single measure of the PHQ-9 when used, for example, to screen for depression ^44^. Likewise, the good reliability of the PHQ-9 in Quechua is in agreement with the findings of the study in Spanish-speaking Peruvians ^1^, and with what has been observed in countries with a similar sociocultural context such as Chile (α=0.891 and ω=0.896) ^45^, even in other very different sociocultural contexts such as Kenya (α=0.840 and ω=0.840) ^46^.

The PHQ-9 in the three varieties of Quechua have shown measurement invariance, similar to the version for Peruvian Spanish speakers and other versions of the PHQ-9 internationally. In China, invariance was reported according to age and sex groups, as well as strict invariance ^11^. In Kenya, configurational, metric and scalar invariance of the model was determined according to the presence of HIV infection, sex and age groups ^46^. In the United States, the instrument showed configurational, metric and scalar measurement invariance when comparing English-speaking and Spanish-speaking women ^47^, as well as when comparing college students by age group and race ^48^. A Norwegian study reported invariance according to the presence or absence of eating disorders in the female population ^15^. A recent systematic review confirmed measurement invariance in at least 18 groups, including those determined by the sociodemographic variables included in this study ^43^.

The present study demonstrates configurational, metrical, scalar and strict invariance according to Quechua variants, gender and place of residence. Thus, our findings suggest that it is possible to make comparisons between Peruvians who speak different Quechua variants, as well as comparisons between men and women and between urban and rural residents. In the same sense, the PHQ-9 in Quechua showed measurement invariance according to age, marital status and educational level, with similar practical implications.

We recognize some strengths and limitations of the present study that should be highlighted. This is the first study of cultural adaptation to Quechua of the PHQ-9, a tool widely used internationally for the assessment of depressive symptoms. This research was carried out in three different regions of Peru, which are usually neglected in the study and care of their mental health. However, since this was a written version, participants were required to have a minimum level of schooling, which is not always possible to find in the target population. Future studies should overcome this barrier in order to reach Quechua speakers who do not yet know how to read or write. Also, although our study adhered to international standards in order to achieve the best possible cultural adaptation, we understand that there are subtle aspects specific to each Quechua-speaking culture that we have not been able to assess regarding their experiences related to the symptoms that universally define depression. Therefore, it is possible that the adapted instrument may not be able to measure some of the depressive symptoms in a completely correct and/or accurate way in regions of Peru where the Spanish-speaking culture still has very little presence. On the other hand, altitude may be associated with the presence of certain depressive symptoms, and therefore its inclusion may be informative (e.g., in an invariance analysis). Unfortunately, we were unable to assess altitude for logistic reasons, so it would be useful for future studies to include this variable. Also, unlike the multigroup CFA, the MIMIC models can only evaluate the invariant intercept models and factorial means. Therefore, for the variables where we applied MIMIC, we assume that the rest of the structural and measurement parameters (e.g., factor loadings, variance/covariance error, variance/covariance factor) are the same across all levels of these variables. Another limitation is related to the change made in item 7, from “reading newspaper” to “listening to radio”. Although this change is intended to improve the cultural adaptation of the instrument, it is possible that it could cause variations in the measurement of symptoms compared to the Spanish version of the PHQ-9. These variations could be caused by the different cultural characteristics of each Quechua-speaking society. Also, the number of participants was different for each department/variety, and the sampling was non-probabilistic, which formally affects the external validity of the estimates. Nevertheless, this is the best estimate of validity/reliability of the PHQ-9 in Quechua available to date.

In terms of public health, this is an important first step towards knowledge and attention to the mental health of historically underserved populations. The three versions of the PHQ-9 in Quechua can be gradually incorporated into national health surveys (e.g. ENDES), and can also be used by community mental health centers in the different regions of the country as a valid and reliable screening tool.

In conclusion, the PHQ-9 adapted to three variants of Quechua (Central, Chanca and Cuzco-Collao) offers a valid, reliable and invariant unidimensional measurement across groups according to Quechua variant, sex, residence (rural/urban), age, marital status and educational level. This invariance confirms that comparisons can be made with the measurements of the three Quechua versions of the PHQ-9 across the aforementioned groups.
