# Supplementary material for: Cultural adaptation to quechua and psychometric analysis of the patient health questionnaire (PHQ-9) in a peruvian population
Source: Rev Peru Med Exp Salud Publica. 2023 Sep 26;40(3):267–77. doi: 10.17843/rpmesp.2023.403.12571 (PMC10959517; doi:10.17843/rpmesp.2023.403.12571)
Supplement: Supplementary material. — Available in the electronic version of the RPMESP. [file rpmesp-40-03-12571-s001.pdf]

PATIENT HEALTH QUESTIONNAIRE (PHQ-9) VERSIONES EN QUECHUA

PHQ-9 Quechua Cuzco-Collao:

	Kay iskay qhipa hunkay (simanakunapi*) hayk'a kutitam nanarqasunki kay nanaykuna …	Mana hayk'aqpas	Wakin p'unchawkunalla	Ashka p'unchawkuna	Yaqa llapa p'unchawkuna
1	Pisi munayñiyuq / kallpayuq ima ruranapaqpas karqanki				
2	Mana kallpayuq karqanki, sinchi llakisqa sasachaykunamanta (depresión nisqa), utaq manañan q'anchay karqanchu kausayñiykipi / yanqallañan kausaranki				
3	Mana puñuyta atirqankichu utaq sinchita puñuy hap'isurqanki / aysarasunki				
4	Sayk'usqa kachkarqanki, utaq pisi kallpayuq				
5	Pisi mikhuyllata munarqanki, utaq achkhata mikuyta munarqanki				
6	Mana allinchu qamwan kakurqanki, musyakunki (sintikunki*) pantaq hina utaq qamtapas pantakuwaq hina hinallataq aylluykitapas (familiaykitapas*)				
7	Sasachaywanchu yuyayta hap'irqanki (pisita umallirqanki), radiota* uyarispa‡ utaq televisionta* qhawaspa				
8	Pisitachu kuyurqanki utaq allillamanta rimarqanki hinaspa wak runakuna ripararqanku, icha anchatachu kuyuqurkanki utaq mana qasichu karqanki, ñisuta purikacharqanki.				
9	Umaykipi wañuy yuyaylla karqan utaq imaynatapis nanachikuyta/k'irikuyta munarqanki.				

*Palabras que combinan quechua con español; ‡ Cambio "leer periódico" por "escuchar radio"

PHQ-9 Quechua Chanca:

N°	Kay iskay qhipa simanakunapi hayk'a kutitam nanasurqanki kay nanaykuna …	Mana hayk’aqpas	Wakin p’unchawkunalla	Ashka p’unchawkuna	Yaqa llapa p’unchawkuna
1	Pisi munayniyuq / kallpayuqchu ima ruranapaqpas karqanki				
2	Mana kallpayuq karqanki, sinchi llakisqa sasachaykunawan (depresión nisqa), utaq manañan allinchu karqanki kawsaynikipi yanqallaña kawsaranki				
3	Mana puñuyta atirqankichu utaq sinchita puñuy hap'isurqanki / aysarasunki				
4	Pisipasqa kachkarqanki, utaq pisi kallpayuq				
5	Pisi mikhuyllata munarqanki, utaq achkhata mikuyta munarqanki				
6	Mana allinchu qanwan tarikurqanki, sintikunki pantaq hina utaq qamtapas pantakuwaq hina, hinallataq aylluykitapas				
7	Sasachakuywanchu pisita umallirqanki, radiota* uyarispa‡ utaq televisionta* qhawaspa				
8	Pisitachu kuyurqanki utaq allillamanta rimarqanki, hinatan wakin runakunapas yuyay qukurunampaq qina, icha anchatachu kuyuqurkanki utaq mana qasichu karqanki, utqayman purikacharqanki.				
9	Umaykipi wañuy munaylla karqa utaq imaynatapas nanachikuyta o k'irikuyta munarqanki.				

*Palabras que combinan quechua con español; ‡ Cambio “leer periódico” por “escuchar radio”

PHQ-9 Quechua Central:

	Kay ishkay qipa simaanakunachaw, ¿ayka kutitaq qishyapaakullarqunki?	Mana imaypis	Wakin hunaqkuna	Atska hunaqkuna	Kasi llapan hunaqkuna
1	Wallkalla hunaq ruraykikunachaw, itsa manapis kushilla ruray.				
2	Mana ruray munaq, allaapa llakishqa, mana kaway munay.				
3	Mana punuy tsariy, allaapa punukuy.				
4	Pishipashqa, utishqa, kallpaanaq kay				
5	Mana mallaqayyuq, allaapa mikukuy.				
6	Kikikiwan mana alli kay, ruray mana patsakaptin, kikin mana allí kaptin aw aylluyki mana alli kaptin.				
7	Radiyu*, tilivisyun* nawintsaychaw‡ alliqlla yarpachakur mana allimpa tinkutsiy.				
8	Yachayllapa muyu/kuyuy wakin runakuna rikaraamunanpaq. Huknawpa, ruraynin allaapa muyutsin.				
9	Wanushqa kaptii imaykapis alli patsanman, mana alli rurakuyta yarpay.				

*Palabras que combinan quechua con español; ‡ Cambio “leer periódico” por “escuchar radio”
