# Supplementary material for: Cultural adaptation to quechua and psychometric analysis of the patient health questionnaire (PHQ-9) in a peruvian population
Source: Rev Peru Med Exp Salud Publica. 2023 Sep 26;40(3):267–77. doi: 10.17843/rpmesp.2023.403.12571 (PMC10959517; doi:10.17843/rpmesp.2023.403.12571)
Supplement: Translation

# Translation: Adaptación cultural al Quechua y análisis psicométrico del Patient Health Questionnaire (PHQ-9) en población peruana

**Authors:** Julio Cjuno, Félix Julca-Guerrero, Yulisa Oruro-Zuloaga, Frinee Cruz-Mendoza, Admirson Auccatoma-Quispe, Heber Gómez Hurtado, Frank Peralta-Alvarez, Juan Carlos Bazo-Alvarez

**Affiliations:** 1 Universidad Cesar Vallejo, Escuela de Medicina, Piura, Perú.; 2 Universidad Nacional Santiago Antúnez de Mayolo, Huaraz, Perú.; 3 Universidad Peruana Unión, Escuela Profesional de Psicología, Lima, Perú.; 4 Escuela de Educación superior pedagógico José Salvador Cavero Ovalle, Ayacucho, Perú.; 5 Institución Educativa N° 38848 Pucara-Uchuraccay, Ayacucho, Perú.; 6 Universidad Tecnológica del Perú, Chimbote, Perú.; 7 Instituto de Investigación, Capacitación y Desarrollo Psicosocial y Educativo PSYCOPERU, Chimbote, Perú.; 8 Universidad Sedes Sapientiae, Lima, Perú.; 9 Universidad Privada Norbert Wiener, Lima, Perú.; 10 Research Department of Primary Care and Population Health, University College London, Londres, Reino Unido.

**Keywords:** Cuestionario de Salud del Paciente, Depresión, Pueblos Indígenas, Psicometría

## Abstract

**Objetivo:**

. Traducir y adaptar culturalmente el Patient Health Questionnaire (PHQ-9) a tres variedades del quechua y analizar su validez, confiabilidad e invarianza.

**Materiales y métodos:**

. 1) Fase de adaptación cultural: el PHQ-9 fue traducido del inglés a tres variantes del quechua (Central, Chanca, Cuzco-Collao) y traducido nuevamente al inglés, posteriormente expertos y grupos focales permitieron adaptar culturalmente las traducciones. 2) Fase psicométrica: se evaluó la uni-dimensionalidad del PHQ-9 adaptado mediante un Análisis Factorial Confirmatorio (CFA), la confiabilidad se evaluó mediante consistencia interna (Alpha y Omega), y la invarianza de medida según variedades del quechua y variables sociodemográficas se evaluó empleando CFA multigrupos y modelos MIMIC (Múltiples Indicadores y Múltiples Causas).

**Resultados:**

. Cada una de las adaptaciones del PHQ-9 a las tres variedades de quechua reportaron ítems claros y culturalmente equivalentes. Posteriormente, con 970 datos de quechuahablantes adultos varones y mujeres, el modelo general unidimensional reportó un ajuste adecuado (índice de ajuste comparativo: 0,990, índice de Tucker-Lewis: 0,987, residuo estandarizado cuadrático medio: 0,048, raíz del error cuadrático medio de aproximación: 0,071), lo mismo ocurrió para cada variedad del quechua. La confiabilidad fue alta para todas las variedades (α = 0,865 - 0,915; ω = 0,833 - 0,881). Los resultados del CFA multigrupos y modelos MIMIC confirmaron invarianza de medida según variante del quechua, sexo, residencia, edad, estado civil y nivel educativo.

**Conclusiones:**

. Las adaptaciones del PHQ-9 a Quechua Central, Chanca y Cuzco-Collao ofrecen una medición válida, confiable e invariante, confirmando que se pueden hacer comparaciones en los grupos evaluados. Su uso beneficiará a la investigación y a la atención en salud mental de poblaciones quechuahablantes.

## INTRODUCCIÓN

La depresión es un trastorno mental común, cuyas causas incluyen interacciones complejas entre factores sociales, psicológicos y biológicos ^1^. Según el reporte de la Organización Mundial de la Salud, durante el 2021 la depresión estuvo presente en más del 5% de adultos de la población mundial ^2^, incrementándose en un 25% más durante la pandemia por COVID-19^3^. En Perú, de 57 446 encuestados, el 60,1% de personas con algún diagnóstico (n=9383) de salud mental que vivían en la sierra y costa peruana a excepción de Lima Metropolitana presentaron síntomas depresivos durante la pandemia ^4^. Otro estudio peruano, que evaluó a 31 996 participantes, encontró que vivir en la región de la sierra era un factor importante para el incremento de síntomas depresivos clínicamente relevantes, reportando 9,0% de casos moderados a severos, frente a un 5,8% en otras regiones ^5^.

El Patient Health Questionnaire (PHQ-9) es un instrumento psicométrico diseñado para evaluar síntomas depresivos según los criterios del *Diagnostic and Statistical Manual of Mental Disorders - IV*^6^. Originalmente escrito en inglés con un solo factor ^7^, es muy usado en la práctica clínica e investigación a nivel internacional ^8^. Cuenta con numerosas adaptaciones en más de 18 idiomas en 24 países ^9^, tales como francés ^10^, chino mandarín^11^, español ^12^, ruso ^13^, alemán ^14^, noruego ^15^, persa ^16^, lituano ^17^^)^ e incluso kinyarwanda ^18^. La versión en español de este instrumento ha mostrado buenas propiedades entre hispanohablantes del Perú ^1^.

En un estudio previo sobre la versión peruana del PHQ-9 (en español) ^1^, reportamos evidencias de invarianza de medida según variables socio-demográficos: sexo, edad, nivel educativo, nivel socioeconómico, estado civil y área de residencia (rural/urbano). Dicha evidencia permite realizar comparaciones entre diferentes grupos (según las categorías de estas variables), garantizando que las mediciones del PHQ-9 señalan una experiencia subyacente de síntomas depresivos ‘similar a través de estos grupos’ (v.g., hombres y mujeres) ^19^. Esta evaluación de invarianza no se ha realizado aún para ninguna versión del PHQ-9 en quechua. Sin el análisis de invarianza, no hay una garantía de que el PHQ-9 mida la depresión de la misma manera en todos los grupos de quechuahablantes, de allí su necesidad ^20^.

No obstante, aún no se tiene una versión en quechua del PHQ-9 para población peruana. El Perú cuenta con 3 799 780 habitantes que tienen al quechua como primera lengua, lo que representa un 13,6% de la población censada en 2017 ^21^. Las personas autoidentificadas étnicamente como quechuas pueden percibir y expresar los síntomas depresivos de forma diferente a un hispano hablante lo que dificulta el uso del PHQ-9 en español. Por otro lado, el quechua es una familia de lenguas y presenta mucha variación dialectal ^22^. Las variedades mayores por el número de hablantes y extensión geográfica son: quechua Central (QC), quechua Ayacucho-Chanka (QAC) y quechua Cuzco-Collao (QCC), los cuales presentan muchas diferencias ^23^^,^^24^. En lo fonológico, el QC tiene 24 fonemas, el QAC tiene 19 fonemas y el QCC cuenta con 28 fonemas. En lo morfológico, por ejemplo, la acción progresiva y la primera persona actora ‘estoy yendo’; en el QC se marca con -yka y -V (aywa-yka-a), en QAC se marca con -chka y -ni (ri-chka-ni) y en QCC se marca con -sha y -ni (ri-sha-ni). Asimismo, en lo léxico, las palabras ‘amarillo’ y ‘acelerar’ en el QC corresponden a qallwash y wip, en QAC hillu y utqay y en QCC q’illu y utqhay, respectivamente ^24^. Así, la variación lingüística es amplia y; consecuentemente, la intercomprensión mutua entre los hablantes de las diversas variantes del quechua es muy difícil y casi imposible, por lo que no se podría realizar una encuesta única usando solo una de las variedades. La solución *ad hoc* típicamente adoptada por usuarios locales consiste en asistirse de una tercera persona bilingüe que “traduzca” las preguntas y respuestas al cuestionario (v.g., un familiar más joven con una cosmovisión distinta a la persona evaluada). Sin embargo, esta práctica no es recomendable pues introduce ruido en la evaluación ^25^. En Perú, la mayor parte del personal de salud no domina el quechua como primer o segundo idioma, tal y como lo menciona Montesinos-Segura *et al*. ^26^ en la discusión de su estudio. En tal sentido, una apropiada evaluación de los síntomas depresivos realizada por el personal de salud a la población general quechua-hablante es muy difícil en la actualidad, dada esta limitación inherente al idioma.

Por lo tanto, el objetivo de nuestro estudio fue desarrollar la traducción y adaptación cultural del PHQ-9 para tres variedades del quechua, y analizar la validez de estructura interna, confiabilidad e invarianza de medida (por variantes del quechua y variables sociodemográficas) del instrumento adaptado.

**MENSAJE CLAVE**

Motivación para realizar el estudio. Perú es el país con mayor población quechuahablante en Sudamérica, pero no cuenta con un instrumento para evaluar la depresión adaptado culturalmente a poblaciones quechuas.

Principales hallazgos. Se obtuvo una versión del PHQ-9 válida y confiable para ser usada en poblaciones quechuahablantes de la variedad Central, Chanca y Cuzco-Collao.

Implicancias. Esta nueva versión podrá implementarse en las encuestas de salud nacionales y centros de salud mental comunitarios para tamizaje y evaluación de síntomas depresivos.

## MATERIAL Y MÉTODOS

### Diseño y contexto

Estudio instrumental ^27^, desarrollado en cuatro departamentos del Perú (Ancash, Ayacucho, Puno y Cuzco) según la variedad del quechua. Para evaluar la variedad Cuzco-Collao, seleccionamos adultos de los departamentos de Puno y Cuzco, ubicados al suroeste del territorio peruano. Puno cuenta con 538 127 (57,0%) y Cuzco con 709 892 (74,7%) quechuahablantes ^28^; ambos departamentos se caracterizan por desarrollar actividades comerciales, ganadería y turismo por estar en lugares históricos y arqueológicos. Para evaluar la variedad de quechua Chanca, seleccionamos a habitantes del departamento de Ayacucho, el que reporta 389 045 (81,2%) quechuahablantes, dedicados mayormente al comercio, ganadería y agricultura ^28^. Para evaluar la variedad del quechua central, seleccionamos a pobladores del departamento de Ancash, el cual cuenta con 289 172 (34,0%) quechuahablantes, dedicados mayormente al comercio, agricultura y turismo ^28^.

### Fase de adaptación cultural

*Traducción*

Los métodos empleados durante el proceso de traducción fueron *forward translation* y *back translation*^27^. El PHQ-9 en su versión original (inglés) fue traducido directamente a cada variedad del quechua (Central, Cuzco-Collao, Chanca). Esto fue realizado por dos traductores independientes, quienes son nativos quechuahablantes de cada variedad y con conocimientos avanzados de inglés. Culminada la traducción para cada variedad, los dos traductores y dos investigadores quechuahablantes nativos se reunieron para discutir sobre las diferencias en las traducciones. Una vez resueltas las discrepancias y unificadas las traducciones, se procedió con la traducción inversa (quechua a inglés) de las tres variantes en quechua del PHQ-9. Esto fue realizado por dos traductores que tenían al inglés como lengua nativa y con conocimiento avanzado del quechua para cada variante. Una vez completada las traducciones inversas, los traductores de cada variante se reunieron con dos investigadores del estudio para verificar la traducción inversa junto con la primera traducción, afinando detalles y brindando su visto bueno a la versión final de las tres versiones.

### Adaptación cultural a contextos quechuas

Aplicando el método Delphi, se elaboró una ficha de adaptación cultural que se encuentra en: https://doi.org/10.5281/zenodo.8312191. Esta ficha incluyó algunas preguntas abiertas para palabras no comunes en quechua como «Depresión» o «Sin esperanzas». También se buscó consultar el cambio de «leer periódico» por «escuchar radio», cambio de las categorías de respuesta del PHQ-9 y sobre la relación entre el PHQ-9 en quechua con el DSM-V para el diagnóstico de depresión mayor. La versión del PHQ-9 en quechua central fue revisada por dos psicólogos quechuahablantes, que tenían mínimo tres años de experiencia en la atención de pacientes quechua hablantes con depresión de las provincias de Ancash; uno de ellos tenía grado de maestro y el otro licenciado en psicología. La versión en quechua Cuzco-Collao fue evaluada por cinco psicólogos quechuahablantes, todos con título profesional y al menos un año de experiencia en la atención de pacientes quechuas con depresión en Puno o Cuzco. La versión en quechua Chanca fue evaluada por dos psicólogos con título profesional y con experiencia de al menos un año en la atención de pacientes quechuahablantes con depresión en las provincias de Ayacucho. La interacción entre cada experto y el equipo de investigación se dio en dos (Central) y cuatro rondas (Cuzco-Collao y Chanca) de correos. Adicionalmente, en cada correo se consultó sobre las mejoras realizadas (v.g. basadas en las recomendaciones de los expertos) empleando indicadores de relevancia, representatividad, claridad y equivalencia cultural en una escala de 0 a 3 (donde 3 fue la mejor calificación). Cuando todos los expertos calificaron con 3 las mejoras en todos los indicadores se realizó una reunión virtual en conjunto con el equipo de investigación, con el objetivo de evaluar las sugerencias y llegar a un consenso.

Posteriormente, se organizó un grupo focal (vía Zoom) para cada variedad del quechua. El moderador para cada variedad del quechua fue un psicólogo quechuahablante con dominio de métodos cualitativos. Las reuniones duraron 60 minutos aproximadamente. Al inicio, se solicitó responder el PHQ-9 en quechua mediante una versión en línea compartida vía formulario de Google. Luego, el moderador invitó a los participantes a opinar sobre la claridad y comprensión de los ítems en un lenguaje habitual y sencillo para el quechuahablante. Para la variedad Cuzco-Collao se contó con la participación de cinco personas quechua hablantes (tres mujeres y dos hombres), para el Quechua Chanca participaron cuatro personas (dos mujeres y dos hombres), para el Quechua Central participaron cuatro adultos (tres hombres y una mujer). Para las tres variedades del quechua se tomó en consideración que los participantes sean bilingües (hablen quechua y español) y mayores de 18 años (Figura 1). Los participantes del grupo focal brindaron una opinión favorable respecto a la claridad y comprensión del PHQ-9 en las tres variantes de quechua, confirmado que esta adaptación es clara y acorde a su contexto cultural.

**Figura 1:** Diagrama de flujo de la traducción y adaptación cultural del PHQ-9.

### Fase de análisis psicométrico

*Participantes*

Se aplicó la versión final de la fase anterior a una muestra de 970 adultos seleccionados por muestreo no probabilístico por conveniencia, asegurándonos que para cada variedad del quechua contásemos con no menos de 200 participantes. Este número es incluso mayor que el estándar recomendado para análisis factoriales (20 participantes por ítem y teniendo en cuenta que son 9 ítems, el tamaño mínimo sería 180 participantes) y reportado como adecuado en estudios de simulaciones ^29^. Asimismo, cumple con el tamaño mínimo calculado para el análisis factorial (n=218, ver: https://doi.org/10.5281/zenodo.8312191). Seis encuestadores participaron en la recolección de datos, quienes presentaron el estudio a los participantes y una vez obtenido el consentimiento informado solicitaron completar la encuesta en un formulario de Google. La recolección se llevó a cabo en aquellos pueblos y ciudades con mayor presencia quechua. Se incluyeron a hombres y mujeres, mayores de 18 años, que viven en entornos urbanos y rurales, ubicados en tres regiones (según variante del quechua). Para evaluar la variante Cuzco-Collao incluimos habitantes de los departamentos de Puno y Cusco (n=525), para la evaluar la variante Chanca incluimos habitantes de Ayacucho (n=226), y para el analizar la variante de quechua Central incluimos habitantes del departamento de Ancash (n=219). Todos los participantes eran quechuahablantes bilingües (quechua y español), con una formación académica suficiente como para leer en quechua (v.g. primaria incompleta como mínimo). Se excluyeron a aquellos que hablaban una variedad de quechua diferente a las tres estudiadas.

*Instrumento*

El PHQ-9 consta de nueve ítems que se corresponden con síntomas depresivos del DSM-IV ^6^. Sus opciones de respuesta evocan la frecuencia de aparición de tales síntomas en las últimas dos semanas, considerando la siguiente escala tipo Likert: 0=para nada, 1=varios días (1-6 días), 2=la mayoría de los días (7-11 días), 3=casi todos los días (12 días a más). Luego de aplicar el cuestionario se obtiene una puntuación cruda entre 0 y 27. La versión en español del PHQ-9 ha demostrado validez (v.g., buena bondad de ajuste como medida unidimensional: CFI [índice de ajuste comparativo] = 0,936; RMSEA [raíz del error cuadrático medio de aproximación]= 0,089; SRMR [residuo estandarizado cuadrático medio]= 0,039), así como adecuada confiabilidad (α=ω = 0,87) en población peruana hispanohablante ^1^.

*Covariables*

Fueron utilizadas para caracterizar a la población, así como para estudiar la invarianza de medida del modelo unidimensional según edad (en años), sexo (femenino, masculino), educación (primaria incompleta, primaria completa, secundaria incompleta, secundaria completa, superior incompleta, superior completa), estado civil (soltero, casado/conviviente, divorciado/separado, viudo) y lugar de residencia (rural/urbano).

*Procedimiento*

Para la recolección de datos, se capacitó en el uso y aplicación del instrumento a un encuestador para la variedad de quechua Central, dos encuestadores para el quechua Chanca y tres encuestadores para la variedad del quechua Cuzco-Collao. Todos los encuestadores fueron estudiantes de psicología del tercero o cuarto año de estudios. Los encuestadores identificaron grupos de WhatsApp de padres de familia de instituciones educativas, iglesias cristianas y grupos de asociaciones de comunidades campesinas a quienes presentaron la encuesta en formulario de Google. La recolección de datos inició en febrero y terminó en junio del 2022.

*Análisis estadístico*

Se realizó el análisis descriptivo mediante frecuencias relativas y absolutas para las variables de caracterización ^30^. Posteriormente, se realizó un Análisis Factorial Confirmatorio (CFA, por sus siglas en inglés) del modelo unidimensional (se verificó su unidimensionalidad en Perú en una muestra representativa de 30 449 individuos: https://doi.org/10.5281/zenodo.8312191) utilizando un estimador WLSMV (cuadrados ponderados robustos con media y varianza ajustada), tal y como lo hicimos previamente ^1^. Además, el WLSMV es un estimador insesgado para ítems con respuestas ordinales y distribución no normal ^31^. Reportamos las betas estandarizadas del modelo y las medidas estándar de bondad de ajuste: el chi cuadrado (X^2^) para el modelo versus la línea de base, considerando aceptables valores <3; el CFI, que es adecuado cuando es >0,90; el índice de TLI (índice de Tucker-Lewis), que es aceptable con valores >0,90. Asimismo, el SRMR y la RMSEA, se consideraron adecuados con valores ≤ 0,08 ^32^.

Se evaluó la invarianza de medida a través de grupos definidos por variantes del quechua, sexo y ubicación, mediante un CFA multigrupos. El cambio en el CFI (ΔCFI) y RMSEA (ΔRMSEA) se utilizó como criterio principal para comparar modelos con más restricciones contra modelos con menos restricciones. Los modelos primero asumieron la invarianza configuracional (es decir, estructura factorial similar entre grupos) como modelo base, subiendo a invarianza métrica (es decir, cargas factoriales y estructura factorial similares entre grupos), invarianza fuerte (es decir, umbrales, cargas factoriales y estructura factorial similares entre grupos), e invarianza estricta (es decir, varianzas residuales de elementos, umbrales, cargas factoriales y estructura factorial similares entre grupos). Entre cada modelo, se examinó si ΔCFI < 0,01 o ΔRMSEA < 0,01 para establecer si el modelo más restringido era apropiado ^30^.

Alternativamente, se ajustaron modelos MIMIC (Múltiples Indicadores y Múltiples Causas) para la evaluación de la invarianza de medida según edad, estado civil y nivel educativo (variables para las que el CFA multigrupos no fue viable). Se evaluó la invarianza de los interceptos de los indicadores y las diferencias de medias de las dimensiones latentes, todo a través de grupos según dichas covariables. Hemos preferido usar ∆CFI en lugar de ΔX^2^, porque el primero no se ve afectado por el tamaño de la muestra o la complejidad del modelo ^32^. Para el CFA multigrupos, realizamos un análisis de sensibilidad usando el método recomendado por Yoon y Lai ^33^ para tamaños de muestras disímiles. De hecho, se evalúa cada covariable por separado, comparando para cada una de ellas dos tipos de modelos: 1) una versión saturada donde la covariable explica a todos los ítems observados, pero no a las dimensiones latentes, y 2) una versión del modelo de intercepto invariante donde la covariable explica a todas las dimensiones latentes, pero no a los ítems. De igual forma, se reportan e interpretan los índices de ajuste arriba señalados.

Finalmente, se determinó la confiabilidad empleando el coeficiente Alpha de Cronbach ^34^ y el Omega de McDonald ^35^^)^ . Para garantizar reproducibilidad, los principales códigos del análisis se encuentran en: https://github.com/JCBAZO/R-PHQ9-Quechua. Todos los análisis se realizaron en R Studio versión 4.0.4, con los paquetes “lavaan” ^36^, “lavaan.survey” ^37^ “semTools” ^38^, “semPlot” ^39^^)^ y “Psych” ^40^.

*Consideraciones Éticas*

Este estudio fue evaluado y aprobado por el Comité de Ética de la Facultad de Ciencias de la Salud de la Universidad Peruana Unión con informe n.° 2022-CE-FCS-UPeU-059. El instrumento fue aplicado como auto reporte en formato virtual (formulario de Google), programado para presentar el consentimiento informado primero, por lo que sólo aquellos que aceptaron participar en el estudio desarrollaron la encuesta. Se solicitó la autorización del dueño del copyright del instrumento original (Pfizer) a través de un correo electrónico, quien finalmente autorizó su uso y adaptación.

## RESULTADOS

### Fase de adaptación cultural

Los expertos y el equipo de investigación interactuaron hasta lograr la máxima calificación de adecuación por cada ítem respecto a su adaptación cultural (3 en un rango de 0 a 3), resaltando su relevancia, representatividad, claridad y equivalencia cultural. Adicionalmente, brindaron valiosas sugerencias que se mencionan a continuación.

Algunas recomendaciones contribuyeron a mejorar la adaptación de la palabra *depresión,* la cual inicialmente estuvo traducida como *“llaqui”*. Se sugirió agregar *“sinchi”,* que da una mayor intensidad, quedando *“sinchi llakisqa”* (mucha tristeza) como la mejor comprensión de la depresión (ítem 2). Mientras que para la expresión *sin esperanzas,* los expertos recomendaron implementar la pregunta como *“manañan q’anchay karqanchu kausayñiykipi / yanqallañan kausaranki”* (ítem 2). Por razones de contexto, se cambió la actividad cotidiana *«*leer periódico*»* por *«*escuchar radio*»*, debido a que este último es el medio informativo más utilizado en las tres regiones (ítem 7).

Las opciones de respuesta (tipo Likert) del PHQ-9 traducido también requirieron una reflexión especial por parte de los jueces y participantes del grupo focal. En especial, la categoría *«*casi todos los días*»* presentó problemas de claridad en su traducción. La recomendación final del equipo fue utilizar las expresiones *“Mana hayk’aqpas”, “Wakin p’unchawkunalla”, “Ashka p’unchawkuna”, “Yaqa llapa p’unchawkuna”*, representando el equivalente en español de *“*Nunca*», «*Algunos días*», «*Varios días*», «*Casi todos los días*»*.

### Fase de análisis psicométrico

*Datos de caracterización*

De los 970 participantes bilingües, 560 (57,7%) fueron del sexo femenino, 580 (59,8%) tenían entre 18 y 30 años, 621 (64%) reportaron tener estudios universitarios en proceso o concluidos, 577 (59,5%) eran solteros, y 614 (63,3%) indicaron residir en zona urbana (Tabla 1).

**Tabla 1:** Características de los participantes del estudio.

Características	Total		Central		Chanca		Cuzco-Collao	
	(n=970)		(n=219)		(n=226)		(n=525)	
	n	(%)	n	(%)	n	(%)	n	(%)
Sexo								
Mujeres	560	57,7	122	55,7	141	62,4	297	56,6
Hombres	410	42,3	97	44,3	85	37,6	228	43,4
Edad (años)								
18-30	580	59,8	115	52,5	178	78,8	287	54,7
31-40	166	17,1	41	18,7	22	9,7	103	19,6
41-50	120	12,4	23	10,5	18	8,0	79	15,1
51-68	104	10,7	40	18,3	8	3,5	56	10,6
Educación								
Primaria incompleta	42	4,3	0	0,0	9	4,0	33	6,3
Primaria completa	38	3,9	4	1,8	2	0,9	32	6,1
Secundaria incompleta	40	4,2	8	3,7	8	3,5	24	4,6
Secundaria completa	229	23,6	35	16,0	19	8,4	175	33,3
Superior incompleta	325	33,5	75	34,2	114	50,4	136	25,9
Superior completa	296	30,5	97	44,3	74	32,8	125	23,8
Estado Civil								
Soltero(a)	577	59,5	131	59,8	167	73,9	279	53,1
Casado/conviviente	335	34,5	68	31,1	53	23,4	214	40,7
Divorciado/separado	36	3,7	16	7,3	4	1,8	16	3,1
Viudo(a)	22	2,3	4	1,8	2	0,9	16	3,1
Residencia								
Urbana	614	63,3	155	70,8	144	63,7	315	60,0
Rural	356	36,7	64	29,2	82	36,3	210	40,0

*Validez de estructura interna*

Los tres instrumentos finales (uno por cada variedad de quechua) fueron validados de forma independiente. El modelo de un solo factor reportó adecuados valores de bondad de ajuste para las tres variedades del quechua (Central, Chanca y Cuzco-Collao), así como para la muestra total (CFI= 0,990; TLI= 0,987; SRMR=0,048; RMSEA= 0,071) (Tabla 2). Por otro lado, el análisis de sensibilidad para el CFA multigrupos reportó resultados similares a los aquí mostrados. El único factor latente del modelo de medida (depresión) cargó un mínimo de λ = 0,57 y un máximo de λ = 0,79 a los ítems del PHQ-9 (Figura 2).

**Tabla 2:** Bondad de ajuste del modelo unidimensional de medida de la PHQ-9 y confiabilidad, total y por variantes de quechua.

Índice de bondad de ajuste	Total (N=970)	Central (n=219)	Chanca (n=226)	Cuzco-Collao (n=525)
χ^2^ (36)	13513	2381	2653	10041
CFI	0,990	0,968	0,998	0,995
TLI	0,987	0,958	0,997	0,994
SRMR	0,048	0,082	0,029	0,042
RMSEA	0,071	0,112	0,048	0,058
Alpha	0,895	0,865	0,877	0,915
Omega	0,861	0,834	0,833	0,881

X^2^: Chi-cuadrado, X^2^(gl): para el modelo versus la línea de base, gl: grados de libertad, CFI: índice de ajuste comparativo, TLI: Índice Tucker-Lewis, SRMR: residuo estandarizado cuadrático medio, RMSEA: raíz del error cuadrático medio de aproximación.

**Figura 2:** Modelo general de medida de la PHQ-9 con betas y errores estandarizados (N=970).

*Confiabilidad*

El PHQ-9 reportó buena confiabilidad para todas las variedades de quechua, con valores de Alfa de Cronbach entre 0,865 y 0,915, y con valores Omega entre 0,833 y 0,881 (Tabla 2).

*Invarianza de medida*

Los resultados del CFA multigrupos confirman la invarianza de medida según variantes del quechua, sexo y lugar de residencia (Tabla 3). El modelo configural presentó un buen ajuste a los datos (CFI = 0,935) para las tres variantes de quechua. Luego de confirmar la invarianza configural, se comprobó la invarianza métrica con ΔCFI = 0,001 y ΔRMSEA=0,007 (ambos <0,01). Luego, para la invarianza fuerte, se obtuvo ΔCFI=0,018 y ΔRMSEA=0,005, siendo al menos este último <0,01, por lo que se concluyó también una invarianza fuerte. Finalmente, se confirmó la invarianza estricta con ΔCFI=0,009 y ΔRMSEA=0,001. Los resultados e interpretación fueron similares para los modelos de invarianza según sexo y residencia (Tabla 3).

**Tabla 3:** Índices de ajuste de las pruebas de invarianza de medida entre grupos según variantes de quechua, sexo y lugar de residencia (N=970).

Variable de agrupamiento	PHQ-9 (Estructura Interna)	Invarianza (Modelo)	X^2^	gl	Valor de p	CFI	Δ CFI	RMSEA	Δ RMSEA
Variante Quechua	Unidimensional	1. Configural	207,1	81	-	0,965	-	0,100	-
		2. Métrica	296,3	97	<0,001	0,964	0,001	0,093	0,007
		3. Fuerte	423,2	131	<0,001	0,947	0,018	0,097	0,005
		4. Estricta	523,9	149	<0,001	0,938	0,009	0,098	0,001
Sexo	Unidimensional	1. Configural	182,0	54	-	0,963	-	0,098	-
		2. Métrica	192,0	62	0,683	0,971	0,008	0,082	0,017
		3. Fuerte	201,3	79	0,539	0,970	0,001	0,074	0,008
		4. Estricta	212,1	88	0,443	0,971	0,002	0,068	0,006
Residencia (urbano/rural)	Unidimensional	1. Configural	180,5	54	-	0,962	-	0,098	-
		2. Métrica	220,7	62	<0,001	0,962	0,000	0,091	0,007
		3. Fuerte	214,3	79	1,000	0,965	0,003	0,078	0,013
		4. Estricta	241,8	88	0,005	0,963	0,002	0,075	0,002

X^2^: Chi-cuadrado, gl: grados de libertad, CFI: índice de ajuste comparativo, RMSEA: raíz del error cuadrático medio de aproximación, Δ: diferencia, X^2^(gl): para el modelo versus la línea de base (Satorra-Betler).

Los resultados de los modelos MIMIC confirmaron la invarianza por edad, estado civil y nivel educativo (Tabla 4). Para todas las variables estudiadas, los valores de CFI y TLI fueron >0,98, mientras que los valores de SRMR y RMSEA fueron <0,08. Asimismo, los valores absolutos de ΔCFI, ΔTLI y ΔRMSEA fueron <0,01.

**Tabla 4:** Bondad de ajuste de los modelos MIMIC para el PHQ-9 (N=970).

Covariable	Modelo	CFI	TLI	RMSEA	SRMR	Δ CFI	Δ TLI	Δ RMSEA
Edad	MIMIC saturado	0,990	0,984	0,071	0,048	-	-	-
	MIMIC de intercepto invariante	0,990	0,987	0,063	0,048	0,000	0,003	-0,008
Estado civil	MIMIC saturado	0,990	0,984	0,070	0,048	-	-	-
	MIMIC de intercepto invariante	0,990	0,987	0,064	0,048	0,000	0,003	-0,006
Educación	MIMIC saturado	0,991	0,985	0,069	0,047	-	-	-
	MIMIC de intercepto invariante	0,990	0,987	0,064	0,047	-0,001	0,002	-0,005

MIMIC: Múltiples Indicadores y Múltiples Causas, CFI: índice de ajuste comparativo, TLI: índice Tucker-Lewis, SRMR: residuo estandarizado cuadrático medio, RMSEA: raíz del error cuadrático medio de aproximación, Δ: Diferencia. Las comparaciones (Δ) se realizaron entre el modelo MIMIC saturado y el modelo MIMIC de intercepción invariante para cada covariable estudiada.

## DISCUSIÓN

Este es el primer estudio sobre la adaptación cultural al quechua del PHQ-9, un instrumento estandarizado utilizado internacionalmente para evaluar síntomas depresivos. Tras completar una traducción de ida y vuelta (inglés-quechua-inglés), considerando tres variantes del quechua, completamos la adaptación cultural con ayuda de jueces expertos y representantes de la población objetivo, todos quechuahablantes. La versión adaptada ofrece una medición unidimensional, confiable e invariante a través de grupos según variante del quechua, sexo, residencia, edad, estado civil y nivel educativo. Dicha invarianza confirma que se pueden hacer comparaciones con las mediciones del PHQ-9 quechua a través de los grupos mencionados. Los instrumentos finales se encuentran en el Material Suplementario.

Esta adaptación cultural abre nuevas posibilidades para la evaluación de la depresión en población quechuahablante peruana, tanto con fines de investigación como clínicos. Previamente, a través de una revisión narrativa realizada con búsqueda en PubMed, Web of Science y Scopus, incluyendo estudios en inglés y/o español, se encontraron siete estudios que evaluaron la depresión en poblaciones quechuahablantes ^41^. Únicamente dos de estos estudios usaron un instrumento traducido y adaptado al quechua, la Hopkins Symptoms Checklist (HSCL-25). Sin embargo, esta adaptación sólo consideró una variante: el quechua ayacuchano ^42^. La distinción de variantes es esencial para poder abarcar un mayor universo de personas en las distintas regiones del Perú, de otro modo, persiste el problema de acudir a una tercera persona que traduzca o interprete las preguntas y respuestas al momento de la aplicación. El PHQ-9 adaptado a tres versiones de quechua reduce la necesidad de dicho apoyo, facilitando la evaluación tanto en investigación (v.g. encuestas de salud tipo ENDES) como en espacios clínicos (v.g. instrumento estandarizado requerido según los criterios diagnósticos DSM-V).

El instrumento adaptado cuenta con propiedades psicométricas similares a las del PHQ-9 original y al validado para peruanos hispanohablantes. En ambos casos, la estructura interna del PHQ-9 se determinó como unidimensional, vale decir, un latente único que representa la depresión y se expresa a través de cada uno de los nueve síntomas evaluados por el instrumento ^1^^,^^7^. A nivel internacional, la evidencia sistemática reciente sostiene el modelo unidimensional a través de diversas culturas ^43^, con una aceptación tal que el debate se centra ahora en definir los puntos de corte más precisos para una medida única del PHQ-9 cuando se le usa, por ejemplo, para el tamizaje de la depresión ^44^. Asimismo, la buena confiabilidad del PHQ-9 en quechua guarda concordancia con los hallazgos del estudio en peruanos hispanohablantes ^1^ y lo observado en países con un contexto sociocultural similar como Chile (α=0,891 y ω = 0,896) ^45^, incluso en otros contextos socioculturales muy diferentes como Kenia (α=0,840 y ω=0,840) ^46^.

El PHQ-9 en las tres variedades de quechua ha mostrado invarianza de medida, tal y como ocurrió con la versión para hispanohablantes peruanos y otras versiones del PHQ-9 a nivel internacional. En China, se reportó invarianza según grupos de edad y sexo, incluyendo la invarianza estricta ^11^. En Kenia, se determinó la invarianza configuracional, métrica y escalar del modelo según presencia de infección por VIH, sexo y grupos de edad ^46^. En Estados Unidos, el instrumento mostró invarianza de medida configuracional, métrica y escalar al comparar mujeres de habla inglesa e hispanohablantes ^47^, así como al comparar estudiantes universitarios según grupos de edad y raza ^48^. En Noruega, se reportó invarianza según presencia o ausencia de desórdenes alimenticios en población femenina ^15^. Una revisión sistemática reciente confirmó la invarianza de medida en al menos 18 grupos, incluyendo aquellos determinados por las variables sociodemográficas incluidas en este estudio ^43^.

El presente estudio demuestra invarianza configuracional, métrica, escalar y estricta, según las variantes de quechua, sexo y lugar de residencia. De esta manera, los hallazgos sugieren que, en la población peruana, es posible realizar comparaciones entre pobladores quechuahablantes de distintas variantes, así como comparaciones entre varones y mujeres y entre pobladores urbanos y rurales. En ese mismo sentido, el PHQ-9 en quechua demostró invarianza de medida según edad, estado civil y nivel educativo, con repercusiones prácticas similares.

Hemos detectado algunas fortalezas y limitaciones del presente estudio que es preciso resaltar. Este es el primer estudio de adaptación cultural al quechua del PHQ-9, una herramienta muy utilizada a nivel internacional para la evaluación de los síntomas depresivos. Esto implicó un despliegue en tres regiones diferentes del Perú, usualmente desatendidas en el estudio y cuidado de su salud mental. No obstante, por tratarse de una versión escrita, los participantes requerían tener una escolaridad mínima indispensable que no siempre es posible encontrar en la población objetivo. Futuros estudios deberán superar esta barrera, para procurar llegar a quechuahablantes que aún no saben escribir o leer. Así también, aunque nuestro estudio se adhirió a los estándares internacionales para lograr la mejor adaptación cultural posible, entendemos que existen aspectos sutiles propios de cada cultura quechuahablante que no hemos podido rescatar sobre sus experiencias respecto a los síntomas que definen universalmente a la depresión. Por lo tanto, es posible que el instrumento adaptado no logre medir algunos de los síntomas depresivos en forma totalmente correcta y/o precisa en regiones del Perú donde la cultura hispanohablante tiene aún muy poca presencia. Por otra parte, la altitud puede estar asociada con la presencia de ciertos síntomas depresivos, y por ello su inclusión puede ser informativa (v.g., en un análisis de invarianza). Lamentablemente, no pudimos evaluar la altitud por razones logísticas, por lo sería útil que futuros estudios incluyan esta variable. Así también, a diferencia del CFA multigrupos, los modelos MIMIC solo pueden evaluar los modelos de intercepto invariante y las medias factoriales. Por ello, para las variables donde utilizamos MIMIC, asumimos que el resto de los parámetros estructurales y de medida (v.g., cargas factoriales, error de varianza/covarianza, factor de varianza/covarianza) son los mismos a través de todos los niveles de estas variables. Otra limitación está relacionada al cambio realizado en el ítem 7, de «leer periódico» por «escuchar radio». Si bien este cambio tiene la intención de mejorar la adaptación cultural del instrumento, es posible que pueda ocasionar variaciones en la medición con respecto a la versión del PHQ-9 en español. Estas variaciones podrían ser ocasionadas por las diferentes características culturales propias de cada sociedad quechuahablante. Asimismo, el número de participantes fue diferente para cada departamento/variedad, y el muestreo fue no-probabilístico, lo que formalmente afecta la validez externa de las estimaciones. No obstante, esta es la mejor estimación de validez/confiabilidad del PHQ-9 en quechua disponible a la fecha.

En términos de salud pública, este es un primer paso importante hacia el conocimiento y atención de la salud mental de poblaciones históricamente desatendidas. Las tres versiones del PHQ-9 en quechua pueden incorporarse paulatinamente en las encuestas de salud nacionales (v.g. ENDES), de la misma forma podrán ser utilizados por los centros comunitarios de salud mental en las distintas regiones del país, como una herramienta de tamizaje valida y confiable.

En conclusión, el PHQ-9 adaptado a tres variantes de quechua (Central, Chanca y Cuzco-Collao) ofrece una medición unidimensional válida, confiable e invariante a través de grupos según variante del quechua, sexo, residencia (rural/urbano), edad, estado civil y nivel educativo. Dicha invarianza confirma que se pueden hacer comparaciones con las mediciones de las tres versiones en quechua del PHQ-9 a través de los grupos mencionados.
